# Experimental Impact of Increasing Circuit Resistance in the Artificial Womb

**DOI:** 10.1002/pd.6802

**Published:** 2025-04-22

**Authors:** Haiyan Cao, Marcus G. Davey, Kathleen Young, Zhiyun Tian, Seitaro Kosaka, Maria F. Varela, Alan W. Flake, Jack Rychik

**Affiliations:** ^1^ Department of Surgery Center for Fetal Research The Children's Hospital of Philadelphia Philadelphia Pennsylvania USA; ^2^ Fetal Heart Program Cardiac Center at The Children's Hospital of Philadelphia Philadelphia Pennsylvania USA; ^3^ Department of Ultrasound Medicine Union Hospital Tongji Medical College Huazhong University of Science and Technology Wuhan China; ^4^ Vitara Biomedical Inc. Philadelphia Pennsylvania USA; ^5^ Division of Cardiology, Department of Pediatrics Perelman School of Medicine of University of Pennsylvania Philadelphia Pennsylvania USA

**Keywords:** cardiovascular system, extrauterine environment for neonatal development (EXTEND), hemodynamic change, preterm, ultrasound

## Abstract

**Objective:**

To determine the impact of controlled incremental changes in circuit blood flow resistance to the oxygenator on hemodynamics in the fetal lamb supported by the EXTrauterine Environment for Neonatal Development (EXTEND) system.

**Methods:**

A prospective study of oxygenator circuit clamping was conducted on 7 lambs on EXTEND. Echocardiography was performed at four levels of circuit flow (250, 225, 200 and 300 mL/kg/min). Hemodynamic parameters including cardiac output, velocities and pulsatility indexes (PI) were measured, and physiological parameters were documented.

**Results:**

As circuit resistance increases, combined cardiac output (CCO) declines significantly, with reduction most profoundly evident in the right ventricle with left ventricle flow preserved until reaching the lowest circuit flow level of 200 mL/kg/min. Umbilical artery (UA), umbilical vein (UV), and inferior vena cava (IVC) velocities decrease while middle cerebral artery (MCA) velocities increase. UA PI values change commensurately with changes in circuit resistance; however, MCA PI values did not change. Blood pressure and oxygen extraction elevate with increased circuit resistance, while heart rate and oxygen consumption remain unchanged.

**Conclusions:**

Fetal sheep supported by the EXTEND system undergo hemodynamic and physiological changes in response to alterations in circuit flow, reflecting physiological adaptations to maintain circulatory homeostasis.

**Summary**

- What's already known about this topic?
  ◦ Physiologic extrauterine support with growth and organ maturation is possible in the fetal lamb model. The EXTrauterine Environment for Neonatal Development (EXTEND) system can provide up to 4 weeks of extrauterine support for fetal lambs.
  ◦ The stability of the EXTEND system allows for introduction of stressors in a controlled manner, creating unique opportunities to explore their impact on a host of developing biological systems.
- What does this study add?
  ◦ In the artificial womb, fetal sheep are capable of adapting to varying circuit flow conditions in a predictable physiological regulatory manner for short experimental periods during early extracorporeal support.
  ◦ Our work provides the basis for manipulations in circuit resistance that may need to be made to create optimal hemodynamic conditions when the technology is brought to human application. It also establishes future experimental models to study abnormal placental vascular resistance that occurs in nature.

## 1 Introduction

Physiologic extrauterine support with growth and organ maturation is possible in the fetal lamb model [1]. Our group developed the EXTrauterine Environment for Neonatal Development (EXTEND) system, which provides up to 4 weeks of extrauterine support for fetal lambs. The EXTEND system includes a “pumpless” circulation, with blood flow solely driven by the fetal heart. Initial experiments were conducted at a gestational age of 105–111 days, but more recently we have demonstrated successful support in extremely preterm fetal lambs, with gestational age as low as 90–95 days [2]. The stability of the EXTEND system allows for introduction of stressors in a controlled manner, creating unique opportunities to explore their impact on a host of developing biological systems. For example, fetal lambs on EXTEND subjected to sustained hypoxia manifest differences in brain structural maturation and histopathology similar to those seen in human forms of congenital heart disease [3, 4, 5]. In addition, hypoxia affects regional differences in cerebral blood flow [6], myocardial structure [7], as well as lung development [8]. These investigations provide insights into pathophysiological mechanisms impacting fetal health and wellbeing not attainable through study in the natural state.

In order to efficiently optimize gas exchange, nature has created conditions in which the placenta serves as the lowest point of fetal circulatory vascular resistance [9]. For lambs sustained on the EXTEND system, gas exchange occurs through the mechanical oxygenator, with its circuit also designed to offer very low resistance to flow, one of the key elements contributing to the success of this pumpless system. Excessively high or low circulatory placental vascular resistance can result in deleterious effects, potentially influencing fetal growth, and even threatening fetal survival. In our EXTEND experiments, serial echocardiography provides for monitoring of hemodynamic changes at baseline as well as during introduction of various stressors [10]. In the clinical human arena, placental blood flow is characterized through Doppler evaluation of the umbilical artery and umbilical vein [9]. These measures as well as numerous other cardiovascular parameters are also obtainable in fetal lambs on EXTEND [11].

The purpose of this study was to determine the impact of controlled changes in circuit blood flow impedance (a surrogate for placental circulatory resistance) through incremental increases in circuit resistance on hemodynamic changes as measured by echocardiography in the fetal lamb on EXTEND. We explore the impact of increasing placental vascular resistance on hemodynamics, establish future experimental models to study abnormal placental vascular resistance that occurs in nature, and provide knowledge with implications for clinical human applications of this technology.

## 2 Methods

### 2.1 Animals and Surgical Procedure

This is a prospective study of fetal lambs sustained on the EXTEND system. Inclusion criterion was gestational age between 90 and 95 days, and survival for a minimum of 3 days on the EXTEND system, with intent to maintain on circuit for up to 28 days in accordance with the clinical protocol. Mean weight of lambs at the time of study was 0.90 ± 0.05 kg. The methodologies for anesthetic and surgical procedures have been described [1]. All procedures were approved by the Institutional Animal Care and Use Committee (IACUC) of the Children's Hospital of Philadelphia (IAC 19‐00984).

### 2.2 EXTEND System With Auto‐Hoffman Control

**FIGURE 1:**
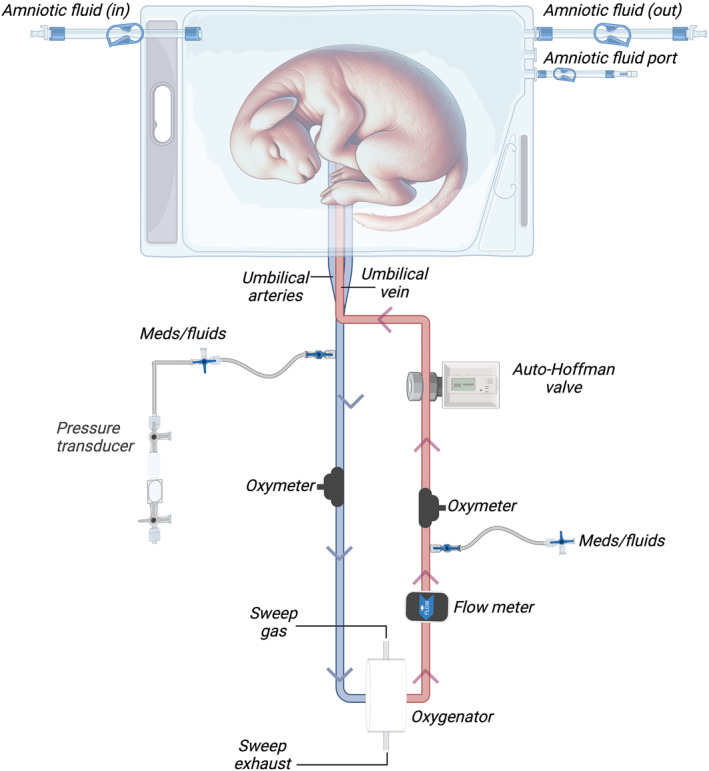
Schematic diagram of the fetal lamb within the EXTrauterine Environment for Neonatal Development (EXTEND) with automatic Hoffman control device (“auto‐Hoffman”). (Created in BioRender. Varela, M. (2024) https://BioRender.com/f04x657).

As a means to prevent significant fluctuations in circuit flow resulting from factors such as strenuous fetal sheep activity or umbilical cord stretching, we incorporated an automatic Hoffman control device (referred to as “auto‐Hoffman”) into our study to ensure the stability of circuit flow. The device is situated at the umbilical vein side of the oxygenator circuit (hereby referred to as the “circuit”), prior to umbilical venous blood returning to the fetal sheep. The “auto‐Hoffman” is comprised of a pinch valve (a position‐adjustable metal valve which regulates circuit flow), and a valve controller (a digital component with a microprocessor unit within and a knob for setting the value of flow). By rotating the knob to a desired setpoint, the “auto‐Hoffman” either increases or decreases resistance by pinching the tubing and thus maintains the stability of the circuit flow at the specified target flow value (Figure 1).

### 2.3 Circuit Clamping Study Protocol

**FIGURE 2:**
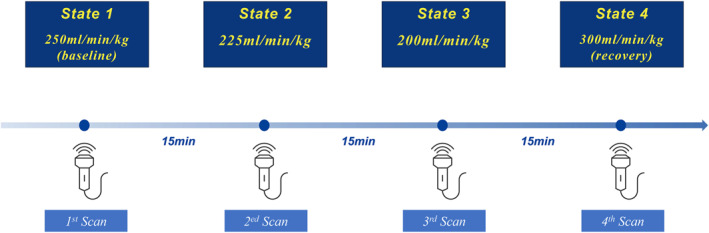
Flowchart of the circuit clamping study protocol.

The circuit clamping experiment was conducted from day 1 to day 3 of the EXTEND support. In the experiment, four distinct levels of circuit flow were established: 250 mL/min/kg (State 1, baseline), 225 mL/min/kg (State 2), 200 mL/min/kg (State 3), and 300 mL/min/kg (State 4, the recovery state). The initial step involved scanning in the baseline state (250 mL/min/kg) for a period of approximately 15 min. Subsequently, the “auto‐Hoffman” was slowly adjusted to increase resistance to the target circuit flow setting (225, 200, 300 mL/min/kg), and with each change, there was at least a 15‐min stabilization period allowed before echocardiographic imaging was performed. In total, 4 echocardiogram examinations were conducted in 4 different flow states. The specific experimental flowchart is presented in Figure 2.

### 2.4 Fetal Echocardiography

All fetal echocardiographic examinations were performed by a single investigator (HC). Images and data measures were acquired using an IE33 ultrasound system (Philips Healthcare, Bothell, WA, USA) equipped with an S8‐3 sector array probe and an L11‐3 linear array probe.

#### 2.4.1 Cardiac Parameters

**FIGURE 3:**
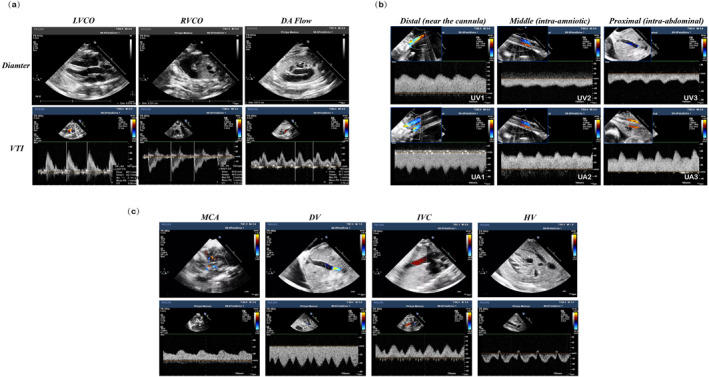
Ultrasonic parameters obtained in fetal lambs on EXTEND support. (a) Measurement of diameters and spectral Doppler waveforms of aortic valve, pulmonary valve, and ductus arteriosus. (b) Spectral Doppler waveforms and measuring sites of the distal (near the cannula tip, UV1/UA1), middle (intra‐amniotic, UV2/UA2), and proximal (intra‐abdominal, UV3/UA3) portions of umbilical artery and vein. (c) Spectral Doppler waveforms and measuring sites of middle cerebral artery (MCA), ductus venosus (DV), inferior vena cava (IVC), and hepatic vein (HV).

Specific echocardiographic parameters collected included diameters and velocity time integrals (VTIs) of aortic valve (AV), pulmonary valve (PV) and ductus arteriosus (DA) and heart rate (HR). Diameters and VTIs of AV, PV, and DA were measured in the left ventricular outflow tract view, right ventricular outflow tract view, and three‐vessel and tracheal views, respectively (Figure 3a). Left ventricular cardiac output (LVCO), right ventricular cardiac output (RVCO), and DA flows are calculated as: (diameter (cm) of AV (PV, DA)/2)^2^ × 3.14 × VTI (cm) × HR/estimated daily weight (kg). Combined cardiac output (CCO) is the sum of LVCO and RVCO. Umbilical flow is characterized through measures of systolic, diastolic, mean velocity, and pulsatility index (PI) of umbilical artery (UA) and umbilical vein (UV). PI is calculated as (peak systolic velocity–end‐diastolic velocity)/mean velocity. Umbilical arterial systolic/diastolic ratio (S/D) is calculated as peak systolic velocity/end‐diastolic velocity.

#### 2.4.2 Umbilical Circuit Parameters

Considering the artificial nature of the pathway to and from the circuit (consisting of cannulae and tubing), we wished to explore for any possible differences in measures obtained sampling at various sites along the umbilical arterial and venous systems. Thus, measurements of velocities and PIs were performed at three different sites of the umbilical cord: 1) distal portion (near the cannula tip, referred to as UA1, UV1), 2) middle portion (intra‐amniotic portion, referred to as UA2, UV2), and 3) proximal portion (intra‐abdominal portion, referred to as UA3, UV3) (Figure 3b).

#### 2.4.3 Cerebral Flow Parameters

We obtained cerebral flow through measures of peak systolic, diastolic, mean velocities, and calculated PI of middle cerebral artery (MCA) (Figure 3c). Cerebroplacental ratio (CPR) was calculated as: MCA PI/UA PI.

#### 2.4.4 Venous Flow Parameters

We obtained venous flow in the ductus venosus (DV), inferior vena cava (IVC) and right hepatic vein (HV) and measured systolic, diastolic, mean velocities, and PI of ductus venosus (DV) flow. We also measured the first peak forward velocity during systole (S), the second peak forward velocity during early diastole (D), and the nadir velocity during atrial contraction (a) of IVC and HV (Figure 3c).

### 2.5 Physiological Data Acquisition

Physiological parameters of fetal lambs were continuously monitored and automatically acquired by the experimental system (LabChart 8, ADInstruments Inc.) as previously reported [1]. Physiological parameters during the echocardiographic scans were averaged over 1 min intervals. Specific parameters included: circuit flow (indexed to body weight), HR, systolic/diastolic/mean blood pressure (Syst/Diast/Mean BP), UV (i.e. post‐oxygenator) oxygen saturation (UV SO_2_), UA (i.e. pre‐oxygenator) oxygen saturation (UA SO_2_). UA and UV SO_2_ difference was calculated as: UV SO_2_—UA SO_2_. Body weight of the fetal sheep was recorded on the surgery day (Day 0), and then calculated as a daily increase of 20 g/kg/day, as previously described [1]. Oxygen metabolic parameters included oxygen delivery, oxygen consumption, and oxygen extraction. Oxygen delivery (mL kg^−1^ min^−1^) was calculated as = [weight indexed circuit flow × post oxygenator membrane oxygen content]; oxygen consumption (mL kg^−1^ min^−1^) as = [weight indexed circuit flow × (post oxygenator membrane oxygen content—pre oxygenator membrane oxygen content)]; oxygen extraction (%) as = (oxygen consumption/oxygen delivery) × 100%, with oxygen content = [(1.34 × Hgb × oxygen saturation) + (0.0031 × PaO_2_)].

### 2.6 Statistical Analysis

Continuous variables are expressed as mean ± standard deviation (SD). The normality of the data was assessed using the Shapiro‐Wilk test. To examine differences in parameters across different levels of circuit flow, repeated‐measures ANOVA was employed for normally distributed data, while Friedman test was utilized for non‐normally distributed data. For comparisons between different states (i.e., State 1 vs. 2; State 2 vs. 3; State 1 vs. 3; State 3 vs. 4), we utilized paired *t*‐tests (for normally distributed data) and Wilcoxon signed‐rank test (for non‐normally distributed data).

For comparative analysis of PIs and velocities in 3 different segments of the umbilical artery and vein, we employed one‐way ANOVA with a Fisher's least significant difference (LSD) *post‐hoc* test (for normally distributed data) and Kruskal‐Wallis (K‐W) test (for non‐normally distributed data). All data were analyzed using SPSS version 27.0 (IBM; Armonk, New York, USA) and GraphPad Prism version 10.1.2. The level of statistical significance was set at a two‐sided *p*‐value of less than 0.05.

## 3 Results

### 3.1 Study Group Overview

The circuit clamping study was conducted on 7 individual fetal lambs (2 female, 5 male) at a gestational age of 92–95 days (Table 1). Mean body weight at cannulation, and at study was 0.86 ± 0.05 kg, and 0.90 ± 0.05 kg, respectively. Mean survival days for the 7 animals was 16.43 days (range, 4–26 days). No significant technical complications were observed during the cannulation procedure, the transition to EXTEND, or during the circuit clamping study. Death in animals # 2 and # 4 occurred prematurely days after our experiments due to cord spasm and bleeding complications and were unrelated to cardiac dysfunction or high‐output cardiac failure.

**TABLE 1:** Summary of experimental animals.

Number	1	2	3	4	5	6	7	Mean ± SD
Gestational age at study, days	95	92	94	94	95	93	95	94.00 ± 1.15
Days on EXTEND when study performed, days	3	1	2	1	3	2	3	2.14 ± 0.90
Weight at study, kg	0.91	0.80	0.93	0.94	0.86	0.93	0.91	0.90 ± 0.05
Weight at cannulation, kg	0.86	0.78	0.89	0.92	0.81	0.89	0.86	0.86 ± 0.05
Gender	F	M	M	F	M	M	M	
Size of arterial cannulas, Fr	12/12	12/10	12/12	12/12	12/10	12/12	12/10	
Size of venous cannula, Fr	14	14	14	14	14	14	14	
Length of run, days	22	7	26	4	19	17	20	16.43 ± 8.02

Abbreviations: EXTEND, EXTrauterine Environment for Neonatal Development; F, female; M, male.

### 3.2 Cardiac Output

**FIGURE 4:**
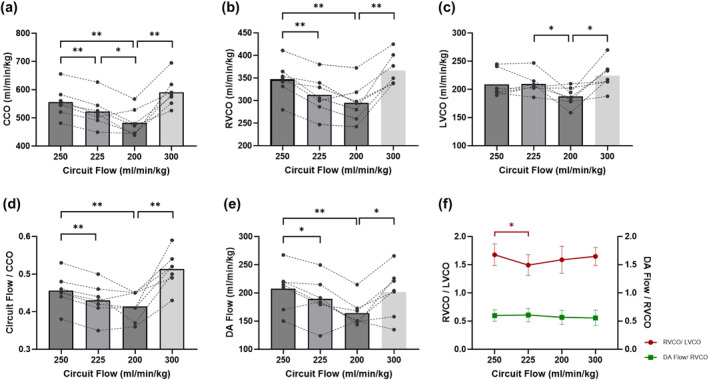
Cardiac hemodynamic changes in circuit clamping study. (a) Cardiac output (CCO). (b) Right ventricular cardiac output (RVCO). (c) Left ventricular cardiac output (LVCO). (d) The ratio of circuit flow to CCO. (e) Ductus arteriosus (DA) flow. (f) The ratio of LVCO to RVCO (left vertical axis), and the ratio of DA flow to RVCO (right vertical axis).

2Increase in resistance via tightening of the auto‐Hoffman clamp from baseline to State 2 results in a decline in CCO, with a reduction from 556 ± 54 mL/min/kg at baseline to 522 ± 55 mL/min/kg (*p* < 0.001). This decline continues, with a further reduction to 482 ± 48 mL/min/kg at State 3 (*p* = 0.028). During the recovery state (State 4), there is an increase in CCO observed in comparison to State 3 (591 ± 54 vs. 482 ± 48 mL/min/kg, *p* < 0.001). The ratio of circuit flow to CCO is 0.46 ± 0.04 at baseline, but exhibits a decline to 0.43 ± 0.05 at State 2 (*p* < 0.001), and 0.41 ± 0.04 at State 3 (*p* = 0.009) (Figure 4a,d; Supporting Information S1: Table S1).

Notably, the changes in flow were not consistent between RVCO and LVCO. A reduction in circuit flow from baseline 250 to State 2225 mL/min/kg results in a decline in RVCO, from 347 ± 39 mL/min/kg at baseline to 312 ± 42 mL/min/kg (*p* = 0.002), whereas LVCO remains relatively stable (209 ± 24 vs. 210 ± 19 mL/min/kg, *p* = 0.872). When resistance is further increased and circuit flow lowered from State 2225 to State 3200 mL/min/kg, a statistically significant decrease in LVCO is observed (210 ± 19 vs. 187 ± 17 mL/min/kg, *p* = 0.046), while RVCO does not decrease significantly (312 ± 42 vs. 295 ± 42 mL/min/kg, *p* = 0.073). Upon restoration of the circuit flow to State 4300 mL/min/kg, both RVCO and LVCO exhibited a significant increase in rebound (all *p* < 0.05). The ratio of RVCO to LVCO at varying circuit flows fluctuated between 1.49 ± 0.18 and 1.67 ± 0.20. Except for a slight decline observed at State 2 (*p* = 0.047), no statistically significant alterations were discerned in the remaining states (Figure 4b,c,f; Supporting Information S1: Table S1).

Alterations in DA flow tracked similarly to RVCO flow. DA flow is 207 ± 38 mL/min/kg at baseline and reached a nadir of 164 ± 25 mL/min/kg at State 3 (*p* = 0.005). The ratio of DA flow to RVCO is stable throughout all conditions within a range of 0.55–0.60 (Figure 4e; Supporting Information S1: Table S1).

### 3.3 Umbilical Circuit Flow

In the circuit of the EXTEND system, the proximal part (UA3) of the UA exhibits significantly elevated PI and S/D values compared to the distal and middle parts. Conversely, the PI of UV demonstrates a relatively consistent pattern across different segments. The proximal segment (UA3) exhibited the highest systolic and mean velocity, whereas the highest velocities of UV were observed in the distal segment (UV1) (Supporting Information S1: Table S2).

**FIGURE 5:**
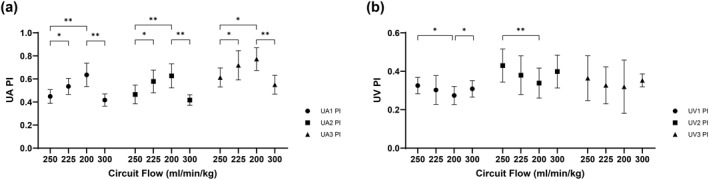
Umbilical flow changes in circuit clamping study. (a) Pulsatility index (PI) of umbilical artery (UA) in distal (UA1), middle (UA2) and proximal (UA3) segments. (b) Pulsatility index (PI) of umbilical vein (UV) in distal (UV1), middle (UV2) and proximal (UV3) segments.

Increased resistance with circuit clamping reveals a discernible increase in the PI and S/D across all UA segments in response to incremental tightening of the Hoffman clamp, followed by a decline during the recovery state (all *p* < 0.05 in comparisons between States 2 vs.1, 3 vs.1, and 4 vs. 3). The majority of UA segments presented a downward trend in velocity, although some of these reductions were not statistically significant. These alterations were more pronounced in the distal and middle portions of UA (Figure 5a; Supporting Information S1: Table S1).

Similar to UA, a significant decline in UV flow velocity is readily discernible with an increase in circuit resistance, particularly in the intra‐amniotic and distal segments. In contrast to the elevated PI observed in UA, a reduction in PI is noted in the distal and middle segments of the UV (Figure 5b; Supporting Information S1: Table S1).

### 3.4 Cerebral Flow

**FIGURE 6:**
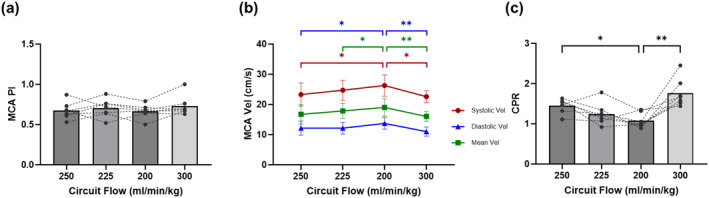
Cerebral flow changes in circuit clamping study. (a) Pulsatility index (PI) of middle cerebral artery (MCA). (b) Systolic, diastolic and mean velocity of MCA. (c) Cerebroplacental ratio (CPR).

During the increase in circuit resistance, MCA PI remains relatively unchanged within a range of 0.66 ± 0.09 to 0.73 ± 0.13 (Figure 6a; Supporting Information S1: Table S1). Reduction in the circuit flow is accompanied by an increase in the MCA peak systolic velocity, whereas a subsequent restoration of the circuit flow leads to a decline in the MCA peak systolic velocity (Figure 6b; Supporting Information S1: Table S1). As mentioned above, there is a notable increase in UA PI while MCA PI remains unaltered, thus resulting in a statistically significant decrease in CPR with each incremental increase in circuit resistance and decrease in circuit flow (Figure 6c; Supporting Information S1: Table S1).

### 3.5 Venous Flow

**FIGURE 7:**
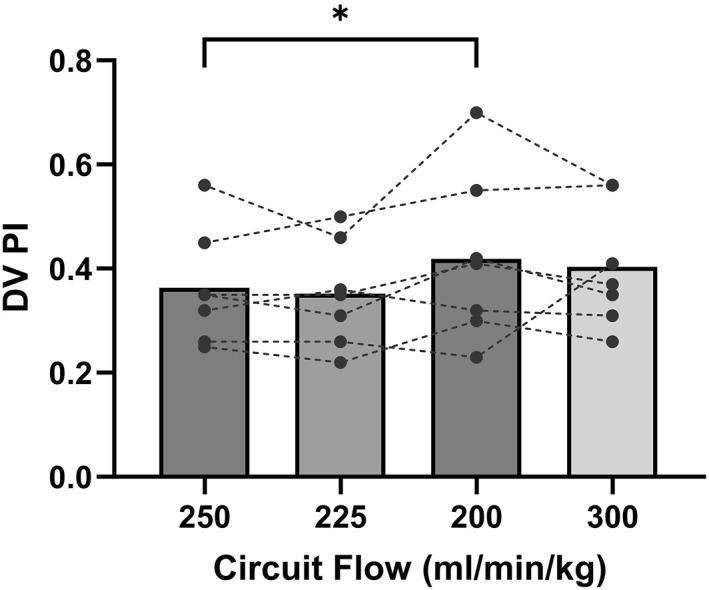
Venous flow changes in circuit clamping study. Pulsatility index (PI) of ductus venosus (DV).

Only when the circuit flow is decreased to 200 mL/min/kg is there a statistically significant increase observed in DV PI with a decrease in DV velocities. However, there is no notable alteration during the recovery state (Figure 7; Supporting Information S1: Table S1). Peak velocities of IVC and HV demonstrated no obvious alterations, with the exception of the D‐ and a‐wave velocities in the IVC, which exhibited a pronounced decline at the nadir circuit flow (200 mL/min/kg) in comparison to baseline. During the State 4 recovery state, there is an increase in both IVC and HV velocities (Supporting Information S1: Table S1).

### 3.6 Physiological Parameters

Circuit flow was measured and recorded in real time throughout the fetal echocardiographic scan. To obtain the circuit flow for each animal, flow values were averaged at one‐minute intervals. The mean circuit flow values measured were 253 ± 3, 223 ± 4, 199 ± 2, and 301 ± 4 mL/min/kg, which were all well aligned with the targeted values of 250, 225, 200, and 300 mL/min/kg as set by the auto‐Hoffman as experimental conditions for the study (Supporting Information S1: Table S1).

**FIGURE 8:**
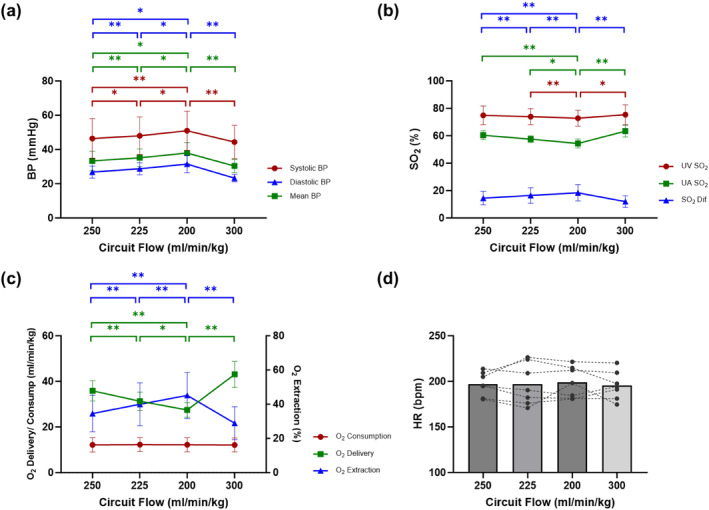
Physiological parameters in circuit clamping study. (a) Systolic, diastolic and mean blood pressure (BP). (b) Oxygen saturation (SO_2_) of umbilical artery (UA) and umbilical vein (UV), and saturation difference between UA and UV (SO_2_ Dif). (c) Oxygen delivery, oxygen consumption and oxygen extraction. (d) Heart rate (HR).

There was no significant change in heart rate (HR) from condition to condition, but notable elevations in blood pressure (BP), which declined below baseline levels during the recovery state (Figure 8a, 8d; Supporting Information S1: Table S1). No statistically significant changes in oxygen saturation (SO_2_) are observed in UA and UV when the circuit flow is reduced to 225 mL/min/kg. However, a notable decline is observed in UA SO_2_ and UV SO_2_ when the flow further declines to 200 mL/min/kg. The difference in UA SO_2_ and UV SO_2_ (between pre and post oxygenator) exhibits a statistically significant increase in conjunction with the gradual decrease in circuit flow (Figure 8b; Supporting Information S1: Table S1). Oxygen consumption remains stable under all circuit flow conditions. Conversely, oxygen delivery decreases significantly with rising Hoffman resistance and decreased circuit flow, resulting in a significant increase in oxygen extraction (Figure 8c; Supporting Information S1: Table S1).

## 4 Discussion

Our study describes the acute physiological response in cardiovascular hemodynamics of EXTEND‐supported fetal lambs when confronted with alterations in circuit flow within the first 1–3 days of support. In the EXTEND system, fetal sheep undergo gas exchange via a mechanical oxygenator, whereas in the natural in‐utero state, gas exchange occurs through the complex structure of the placenta. Whether facing the inspiring design of mammalian evolution, the natural placenta, or our engineered attempt at mechanical replication, the circuit of EXTEND, the fetal cardiovascular system encounters variable loading conditions as it perfuses this essential unit of oxygenation. Circulatory resistance that is either too high, or too low, can have an adverse impact. In nature, when circulatory resistance is elevated, as in placental insufficiency, it results in inadequate blood supply to the fetus and growth restriction [12]. Conversely, when circulatory resistance is abnormally diminished, as in arteriovenous fistulae, it creates a state of volume overload, which may precipitate high‐output heart failure [13]. Anecdotally, during the course of our experiments over the years, we occasionally witnessed several fetal animals that exhibited marked lability in hemodynamics during immediate transition from the natural in utero state onto the EXTEND system right after cannulation, with marked over‐circulation and acute heart failure. We attributed this instability to the fetal cardiovascular system adapting to sudden changes in loading conditions during transition from the natural in‐utero state to an altered vascular resistance load imposed by the circuit. The use of a Hoffman clamp on the circuit during this acute transition allowed us to modulate and temper this response, resulting in improved hemodynamics. Our experiences led us to consider formally exploring the hemodynamic impact of circuit resistance in a controlled manner.

We discovered several findings that lead to considerations of the hemodynamic adaptations that may be taking place. As circuit flow diminishes, so does CCO; however, the right and left sides of the heart differ in their responses. A decrease in RVCO accounts for a greater proportion of the decrease in CCO, and as circuit flow diminishes, LVCO is relatively well maintained. This is consistent with classical research from 1968, which demonstrated that fetal LV and RV perform independently when umbilical venous flow is reduced [14]. Increased Hoffman clamp resistance with reduction in circuit flow reduces umbilical venous return and thus selectively reduces RV preload. The RV is the first ventricle in line for umbilical venous return and is the “dominant” ventricle in fetal life [15]. The fetal heart is geometrically designed such that through a wide‐open ductus arteriosus, the RV is assigned the task of perfusing the lower body and placental circulation, while the LV via connection to the ascending aorta assigns it to perfuse the coronary, upper body and cerebral circulations. The aortic isthmus is the interface bridge between these two systems [16]. We confirmed a direct relationship between changes in RVCO and ductus arteriosus flow in our experiments. Increasing circuit resistance in EXTEND selectively impacts the RV, not just from a preload but also from an afterload perspective. LV output is comparatively, well preserved as a natural adaptation to selectively maintain perfusion of the vital coronary and cerebral circulations. A theoretical supportive finding is the increase in MCA peak systolic velocities seen with increased circuit resistance, suggesting preserved cerebral flow.

As circuit resistance increases, so does the Doppler derived UA PI. This finding strengthens and validates the utility of UA PI as a truly accurate measure of downstream resistance, as it changes with direct changes in the magnitude of Hoffman clamping. Sampling site variability along the UA pathway reveals the same degree of change in UA PI with each incremental experimental change in circuit resistance; however, PI values are all higher when sampled farthest from the oxygenator and closest to the fetus (UA3). This makes sense as it follows Poiseuille's Law with resistance being directly related to length but inversely related to the 4^th^ power of the radius, that is the greater the distance from the circuit, the greater the UA PI values. Establishing consistency in site sampling along the circuit will be of importance as we move toward further study of experimental conditions and clinical applications.

Increasing circuit resistance leads to decreased velocities in the UV, DV, IVC and HV with the exception being the MCA velocities. Interestingly, MCA PI values remain completely unchanged despite changes in circuit resistance with commensurate changes in CCO. This confirms the presence of adaptive regulatory processes at play to preserve flow, likely at the LV output level as stated above, as well as local cerebral autoregulatory mechanisms. Findings of DV changes under experimental conditions are also intriguing. Despite a decrease in overall velocities in the DV with increase in circuit resistance, there is a significant increase in DV PI from baseline to circuit resistance State 3. An increase in DV PI suggests DV constriction in the face of volume depletion, which may also reflect an adaptive regulatory phenomenon to increase streaming across the foramen ovale to selectively improve left‐sided heart filling [17].

Of note, our fetal lambs demonstrated stability despite the various experimental conditions. There were no notable changes in qualitative ventricular function or significant atrioventricular valve regurgitation throughout the study. In real‐time monitoring of the vital signs of the animals, heart rate (HR) remained unaltered with a decrease in circuit flow; however, a statistically significant increase in blood pressure (BP) was noted with each increase in circuit resistance. This suggests a regulatory increase in peripheral vascular resistance, likely in response to decreased CCO, to maintain overall systemic perfusion. Despite this phenomenon, it is interesting to note no changes in HR, and in combination with the echocardiographic findings of normal heart function, suggests little perturbation to the overall well‐being and clinical status of the fetus within the range of circuit resistances tested. This is further confirmed by the oxygen metabolism data. Although increased circuit resistance via Hoffman clamping led to a decrease in oxygen delivery as expected due to reduced circuit flow and decreased CCO, oxygen extraction increased with each phase, and thus oxygen consumption as a reflection of metabolic state remained unchanged (Figure 8c).

There are limitations to our study. This is an acute model in which relatively rapid changes in conditions lead to distinct patterns of cardiovascular response characterized within a short period of time. Whether further adaptive changes might be seen with more sustained alterations over time, for example leaving specific conditions in place for 6, 12, or 24 h, is yet to be determined. As with any animal model with strict experimental conditions, the generalizability and validity of our findings for different conditions or long‐term support should be made with caution. In addition, the magnitude of circuit resistance alteration was relatively modest. More profound changes in circuit resistance may provide information on the limits of hemodynamic response. Echocardiographic examinations were not blinded to the lamb conditional states, hence there is the potential for bias. Unfortunately, we do not have baseline data on the fetal lambs while naturally in‐utero prior to cannulation, which would be of interest to compare to what is created ex utero in EXTEND. Finally, adaptations to the conditions imposed may be species specific or gestationally age based, therefore extrapolation to other animal models and the human may be limited.

## 5 Conclusions

Fetal sheep supported by the EXTEND system undergo hemodynamic and physiological changes in response to alterations in circuit flow to maintain circulatory homeostasis. By establishing an animal model of acute incremental circuit resistance and describing the hemodynamic changes, our study provides the opportunity for the development of a chronic model to further explore the cardiovascular impact of placental insufficiency. Importantly, our study provides practical insight into manipulations that can lead to optimal regulation of circuit flow when the EXTEND system is employed in the preterm human fetus in the near future.

## Ethics Statement

This study was approved by the Institutional Animal Care and Use Committee (IACUC) of the Children's Hospital of Philadelphia (IAC 19‐00984).

## Consent

The authors have nothing to report.

## Conflicts of Interest

Alan Flake and Marcus Davey hold multiple patents related to the EXTEND technology and hold rights to option shares in Vitara Biomedical. Marcus Davey is an employee of Vitara Biomedical. Alan Flake is a paid consultant for Vitara Biomedical and his laboratory is partially funded by a Sponsored Research Agreement (Vitara Biomedical).

## Supporting information

Supporting Information S1

## Data Availability

The data presented in this article are not yet publicly available. Nevertheless, they may be disclosed upon reasonable request to the corresponding author.
