# Supplementary material for: Experimental Impact of Increasing Circuit Resistance in the Artificial Womb
Source: Prenat Diagn. 2025 Apr 22;45(6):795–804. doi: 10.1002/pd.6802 (PMC12137034; doi:10.1002/pd.6802)
Supplement: Supplementary file 1 — Supporting Information S1 [file PD-45-795-s001.docx]

**Supplementary Material**

| **Table S1. Hemodynamic and physiologic changes in circuit clamp study** | | | | | | | | |
| --- | --- | --- | --- | --- | --- | --- | --- | --- |
| ***Parameters*** | ***Mean ± SD*** | | | | ***P-value*** | | | |
|  | ***State 1  (250ml/min/kg)*** | ***State 2 (225ml/min/kg)*** | ***State 3 (200ml/min/kg)*** | ***State 4 (300ml/min/kg)*** | ***State 2* vs. *1*** | ***State 3* vs. *1*** | ***State 3* vs. *2*** | ***State 4* vs*. 3*** |
| ***Cardiac output*** | | | | | | | | |
| CCO, ml/min/kg | 556 ± 54 | 523 ± 55 | 482 ± 48 | 591 ± 54 | **<.001** | **0.002** | **0.028** | **<.001** |
| RVCO, ml/min/kg | 347 ± 39 | 312 ± 42 | 295 ± 42 | 367 ± 35 | **0.002** | **<.001** | 0.073 | **<.001** |
| LVCO, ml/min/kg | 209 ± 23 | 210 ± 19 | 187 ± 17 | 224.33 ± 25 | 0.872 | 0.091 | **0.046** | **0.014** |
| RVCO/LVCO | 1.67 ± 0.20 | 1.49 ± 0.18 | 1.58 ± 0.24 | 1.64 ± 0.16 | **0.047** | 0.349 | 0.254 | 0.552 |
| DA Flow, ml/min/kg | 207 ± 38 | 189 ± 38 | 164 ± 25 | 202 ± 44 | **0.031** | **0.005** | 0.053^a^ | **0.023** |
| DA Flow/RVCO | 0.60 ± 0.11 | 0.61 ± 0.12 | 0.57 ± 0.13 | 0.55 ± 0.14 | 0.563 | 0.299 | 0.237^a^ | 0.830 |
| Circuit Flow/CCO | 0.46 ± 0.04 | 0.43 ± 0.05 | 0.41 ± 0.04 | 0.51 ± 0.05 | **<.001** | **0.009** | 0.181 | **0.001** |
| ***Umbilical artery*** | | | | | | | | |
| **Proximal portion (intra-abdominal)** | | | | |  |  |  |  |
| UA3 PI | 0.61 ± 0.08 | 0.72 ± 0.13 | 0.77 ± 0.10 | 0.55 ± 0.08 | **0.022** | **0.017** | 0.215 | **0.005** |
| UA3 S/D | 1.86 ± 0.17 | 2.12 ± 0.29 | 2.24 ± 0.23 | 1.73 ± 0.14 | **0.017** | **0.011** | 0.240 | **0.003** |
| UA3 Syst Vel, cm/s | 55.43 ± 11.73 | 56.14± 13.06 | 51.00 ± 8.37 | 54.29 ± 13.02 | 0.779 | 0.223 | 0.242 | 0.290 |
| UA3 Diast Vel, cm/s | 30.00 ± 6.83 | 27.14 ± 8.09 | 23.14 ± 5.49 | 31.29 ± 6.24 | 0.170 | **0.014** | 0.104^a^ | **<.001** |
| UA3 Mean Vel, cm/s | 41.57 ± 8.28 | 40.86 ± 9.08 | 36.71 ± 7.83 | 41.43 ± 9.66 | 0.671 | 0.111 | 0.093^a^ | **0.024** |
| **Middle portion (intra-amniotic)** | | | | |  |  |  |  |
| UA2 PI | 0.47 ± 0.08 | 0.58 ± 0.10 | 0.63 ± 0.11 | 0.42 ± 0.04 | **0.014** | **0.007** | 0.344 | **0.007** |
| UA2 S/D | 1.62 ± 0.12 | 1.82 ± 0.19 | 1.95 ± 0.20 | 1.53 ± 0.07 | **0.046**^a^ | **0.005** | 0.208 | **0.004** |
| UA2 Syst Vel, cm/s | 35.00 ± 5.13 | 32.71 ± 6.85 | 32.71 ± 5.79 | 38.71 ± 7.25 | 0.256 | 0.103 | 1.000 | **0.018**^a^ |
| UA2 Diast Vel, cm/s | 21.71 ± 3.73 | 18.29 ± 4.50 | 16.86 ± 3.29 | 25.43 ± 5.19 | **0.007** | **0.017**^a^ | 0.237 | **0.002** |
| UA2 Mean Vel, cm/s | 28.71 ± 4.31 | 25.43 ± 5.59 | 25.42 ± 4.86 | 32.14 ± 6.01 | **0.041** | **0.020** | 1.000 | **0.005** |
| **Distal portion (near cannula tip)** | | | | |  |  |  |  |
| UA1 PI | 0.45 ± 0.06 | 0.54 ± 0.07 | 0.63 ± 0.10 | 0.42 ± 0.05 | **0.016** | **<.001** | 0.059 | **<.001** |
| UA1 S/D | 1.59 ± 0.11 | 1.75 ± 0.16 | 1.97 ± 0.24 | 1.53 ± 0.10 | **0.027** | **<.001** | 0.069 | **<.001** |
| UA1 Syst Vel, cm/s | 44.00 ± 9.24 | 41.00 ± 8.04 | 39.00 ± 6.98 | 48.00 ± 12.32 | **0.049** | **0.043** | 0.318 | 0.072 |
| UA1 Diast Vel, cm/s | 27.57 ± 4.72 | 23.43 ± 4.50 | 20.00 ± 3.83 | 31.57 ± 9.00 | **0.003** | **<.001** | **0.032** | **0.007** |
| UA1 Mean Vel, cm/s | 36.14 ± 6.77 | 32.57 ± 6.73 | 30.00 ± 5.77 | 39.71 ± 10.23 | **<.001** | **0.004** | 0.136 | **0.033** |
| ***Umbilical vein*** | | | | | | | | |
| **Proximal portion (intra-abdominal)** | | | | |  |  |  |  |
| UV3 PI | 0.36 ± 0.12 | 0.33± 0.10 | 0.32± 0.14 | 0.35± 0.03 | 0.485 | 0.354 | 0.861 | 0.571 |
| UV3 Syst Vel, cm/s | 31.29 ± 5.44 | 30.43 ± 8.20 | 25.58 ± 3.41 | 33.71 ± 5.15 | 0.639 | **0.005** | 0.103 | **0.018**^a^ |
| UV3 Diast Vel, cm/s | 21.71 ± 4.31 | 21.43 ± 3.64 | 18.43 ± 2.99 | 23.43 ± 4.08 | 0.818 | 0.090 | 0.121 | 0.060 |
| UV3 Mean Vel, cm/s | 26.71 ± 4.79 | 26.14 ± 6.36 | 22.43 ± 3.21 | 29.14 ± 3.98 | 0.344^a^ | **0.029** | 0.151 | **0.010** |
| **Middle portion (intra-amniotic)** | | | | |  |  |  |  |
| UV2 PI | 0.43 ± 0.09 | 0.38 ± 0.10 | 0.34 ± 0.08 | 0.40 ± 0.09 | 0.196 | **0.006** | 0.268 | 0.209 |
| UV2 Syst Vel, cm/s | 38.43 ± 9.14 | 33.29 ± 9.05 | 29.43 ± 7.55 | 41.86 ± 11.11 | 0.112 | **0.006** | **0.034**^a^ | **0.006** |
| UV2 Diast Vel, cm/s | 24.71 ± 6.02 | 22.86 ± 7.47 | 21.00 ± 5.54 | 27.86 ± 8.63 | 0.495 | **0.021** | 0.343 | **0.047** |
| UV2 Mean Vel, cm/s | 31.71 ± 7.43 | 28.57 ± 8.50 | 25.00 ± 5.60 | 35.43 ± 10.00 | 0.254 | **0.008** | 0.115 | **0.009** |
| **Distal portion (near cannula tip)** | | | | |  |  |  |  |
| UV1 PI | 0.33 ± 0.04 | 0.30 ± 0.07 | 0.27 ± 0.04 | 0.31 ± 0.04 | 0.270 | **0.012** | 0.350 | **0.033** |
| UV1 Syst Vel, cm/s | 66.29 ± 16.78 | 64.43 ± 20.66 | 52.86 ± 12.44 | 83.29 ± 28.86 | 0.668 | **0.004** | **0.019** | **0.005** |
| UV1 Diast Vel, cm/s | 47.29 ± 10.78 | 47.43 ± 15.89 | 40.00 ± 9.38 | 60.57 ± 19.98 | 0.970 | **0.004** | 0.055 | **0.004** |
| UV1 Mean Vel, cm/s | 57.14 ± 14.42 | 56.43 ± 18.74 | 47.14 ± 11.36 | 72.43 ± 24.30 | 0.856 | **0.005** | **0.028** | **0.004** |
| ***Middle cerebral artery*** | | | | | | | | |
| MCA PI | 0.67 ± 0.11 | 0.71 ± 0.11 | 0.66 ± 0.09 | 0.73 ± 0.13 | 0.535 | 0.830 | 0.236 | 0.117 |
| MCA Syst Vel, cm/s | 23.29 ± 3.82 | 24.71 ± 3.30 | 26.29 ± 3.55 | 22.57 ± 1.99 | 0.356 | **0.016**^a^ | 0.234^a^ | **0.017** |
| MCA Diast Vel, cm/s | 12.14 ± 2.34 | 12.14 ± 1.95 | 13.71 ± 1.89 | 11.00 ± 1.53 | 1.000 | 0.062 | **0.042** | **<.001** |
| MCA Mean Vel, cm/s | 16.71 ± 3.20 | 17.86 ± 2.48 | 19.00 ± 2.89 | 16.00 ± 1.63 | 0.339 | **0.016**^a^ | 0.256 | **0.004** |
| CPR | 1.45 ± 0.18 | 1.25 ± 0.27 | 1.08 ± 0.18 | 1.77 ± 0.35 | 0.121 | **0.026** | 0.146 | **0.007** |
| ***Ductus venosus*** | | | | | | | | |
| DV PI | 0.36 ± 0.11 | 0.35 ± 0.10 | 0.42 ± 0.16 | 0.40 ± 0.12 | 0.659 | **0.047** | 0.118 | 0.736 |
| DV Syst Vel, cm/s | 52.00 ± 13.43 | 49.29 ± 8.96 | 48.14 ± 12.48 | 52.43 ± 17.89 | 0.371 | **0.033** | 0.683 | 0.241 |
| DV Diast Vel, cm/s | 36.29 ± 11.94 | 34.14 ± 8.05 | 31.86 ± 10.95 | 35.43 ± 15.40 | 0.329 | **0.018** | 0.224 | 0.232 |
| DV Mean Vel, cm/s | 45.86 ± 12.29 | 43.86 ± 7.67 | 42.14 ± 12.54 | 45.57 ± 16.15 | 0.452 | **0.015** | 0.528 | 0.219 |
| ***Inferior vena cava*** | | | | | | | | |
| IVC S-wave Vel, cm/s | 33.14 ± 7.80 | 31.57 ± 6.11 | 28.71 ± 4.54 | 35.29 ± 5.02 | 0.121 | 0.066^a^ | 0.067 | **0.005** |
| IVC D-wave Vel, cm/s | 22.86 ± 7.24 | 21.29 ± 6.16 | 19.43 ± 5.26 | 26.00 ± 6.81 | 0.130 | **0.025** | 0.168 | **0.007** |
| IVC a-wave Vel, cm/s | 12.00 ± 6.19 | 10.42 ± 4.69 | 4.29 ± 10.44 | 13.57 ± 4.61 | 0.399 | **0.033** | 0.112 | **0.026** |
| ***Hepatic vein*** | | | | | | | | |
| HV S-wave Vel, cm/s | 18.57 ± 2.44 | 19.29 ± 2.75 | 16.57 ± 3.26 | 23.71 ± 5.38 | 0.356 | 0.235^a^ | 0.203^a^ | **0.006** |
| HV D-wave Vel, cm/s | 9.86 ± 1.95 | 11.14 ± 1.68 | 9.00 ± 2.08 | 14.57 ± 5.38 | 0.109^a^ | 0.418 | 0.125 | **0.008** |
| HV a-wave Vel, cm/s | -5.43 ± 4.20 | -7.71 ± 3.09 | -8.14 ± 3.34 | -4.86 ± 5.79 | 0.143 | 0.192 | 0.707 | 0.223 |
| ***Physiological Parameters*** | | | | | | | | |
| Circuit flow, ml/min/kg | 252.50 ± 3.08 | 223.01 ± 4.22 | 198.90 ± 1.88 | 301.24 ± 3.73 | **<.001** | **<.001** | **<.001** | **<.001** |
| Heart Rate, bpm | 197.14 ± 13.11 | 197.01 ± 22.85 | 199.08 ± 17.24 | 195.52 ± 15.68 | 0.980 | 0.688 | 0.670 | 0.488 |
| Syst BP, mmHg | 46.45 ± 11.67 | 48.06 ± 10.97 | 50.94 ± 11.44 | 44.38 ± 9.69 | **0.015** | **0.007** | **0.018**^a^ | **0.002** |
| Diast BP, mmHg | 26.81 ± 3.51 | 28.75 ± 3.51 | 31.50 ± 4.99 | 23.31 ± 2.21 | **0.003** | **0.018**^a^ | **0.018**^a^ | **<.001** |
| Mean BP, mmHg | 33.35 ± 5.69 | 35.19 ± 5.15 | 37.98 ± 6.11 | 30.34 ± 3.86 | **0.003** | **0.018**^a^ | **0.018**^a^ | **<.001** |
| UV SO_2_, % | 74.90 ± 6.78 | 73.96 ± 5.83 | 72.79 ± 5.86 | 75.34 ± 7.23 | 0.303 | 0.060 | **0.008** | **0.031** |
| UA SO_2_, % | 60.41 ± 3.23 | 57.53 ± 2.41 | 54.37 ± 3.22 | 63.36 ± 4.20 | 0.058 | **0.009** | **0.028**^a^ | **0.005** |
| SO_2_ Dif, % | 14.49 ± 4.87 | 16.43 ± 5.63 | 18.41 ± 5.96 | 11.98 ± 4.20 | **0.008** | **0.004** | **0.005** | **0.004** |
| O_2_ Consumption, ml/min/kg | 12.16 ± 3.13 | 12.27 ± 3.07 | 12.21 ± 3.06 | 12.13 ± 3.02 | 0.568 | 0.804 | 0.745 | 0.647 |
| O_2_ Delivery, ml/min/kg | 35.87 ± 4.43 | 31.33 ± 4.01 | 27.45 ± 3.20 | 43.07± 5.72 | **<.001** | **<.001** | **0.018**^a^ | **<.001** |
| O_2_ Extraction, % | 34.54 ± 10.71 | 39.98 ± 12.53 | 45.16 ± 13.46 | 28.89 ± 9.69 | **0.002** | **<.001** | **<.001** | **<.001** |
| CCO, combined cardiac output; RVCO, right ventricular cardiac output; LVCO, left ventricular cardiac output; DA, ductus arteriosus; UA, umbilical artery; PI, pulsatility index; S/D, systolic/ diastolic ratio; Syst, systolic; Diast, diastolic; Vel, velocity; UV, umbilical vein; MCA, middle cerebral artery; CPR, cerebro-placental ratio; DV, ductus venosus; IVC, inferior vena cava; HV, hepatic vein; blood pressure; SO_2_, oxygen saturation.  ^a^ *P*-values according to Wilcoxon signed-rank test. The other *P*-values according to paired *t*-test. (*P* < 0.05, two-tailed) | | | | | | | | |

| ***Table S2*. Comparisons of hemodynamic parameters in different segments of the umbilical artery and vein** | | | | | | | | | | | | |
| --- | --- | --- | --- | --- | --- | --- | --- | --- | --- | --- | --- | --- |
| ***Parameters*** | ***State 1  (baseline, 250ml/min/kg)*** | | | ***State 2  (225ml/min/kg)*** | | | ***State 3  (200ml/min/kg)*** | | | ***State 4  (300ml/min/kg)*** | | |
|  | ***Distal (UA1/UV1)*** | ***Middle (UA2/UV2)*** | ***Proximal (UA3/UV3)*** | ***Distal (UA1/UV1)*** | ***Middle (UA2/UV2)*** | ***Proximal (UA3/UV3)*** | ***Distal (UA1/UV1)*** | ***Middle (UA2/UV2)*** | ***Proximal (UA3/UV3)*** | ***Distal (UA1/UV1)*** | ***Middle (UA2/UV2)*** | ***Proximal (UA3/UV3)*** |
| **UA** |  |  |  |  |  |  |  |  |  |  |  |  |
| PI | 0.45 ± 0.06 | 0.47 ± 0.08 | 0.61 ± 0.08^‡§^ | 0.54 ± 0.07 | 0.58 ± 0.10 | 0.72 ± 0.13^‡^ | 0.63 ± 0.10 | 0.63 ± 0.11 | 0.77 ± 0.10^‡§^ | 0.42 ± 0.05 | 0.42 ± 0.04 | 0.55 ± 0.08^‡§^ |
| S/D | 1.59 ± 0.11 | 1.62 ± 0.12 | 1.86 ± 0.17^‡§^ | 1.75 ± 0.16 | 1.82 ± 0.19 | 2.12 ± 0.29^‡§^ | 1.97 ± 0.24 | 1.95 ± 0.20 | 2.24 ± 0.23^‡§^ | 1.53 ± 0.10 | 1.53 ± 0.07 | 1.73 ± 0.14^‡§^ |
| Syst Vel, cm/s | 44.00 ± 9.24 | 35.00 ± 5.13 | 55.43 ± 11.73^‡§^ | 41.00 ± 8.04 | 32.71 ± 6.85 | 56.14± 13.06^‡§^ | 39.00 ± 6.98 | 32.71 ± 5.79 | 51.00 ± 8.37^‡§^ | 48.00 ± 12.32 | 38.71 ± 7.25 | 54.29 ± 13.02^§^ |
| Diast Vel, cm/s | 27.57 ± 4.72 | 21.71 ± 3.73 | 30.00 ± 6.83 | 23.43 ± 4.50 | 18.29 ± 4.50 | 27.14 ± 8.09 | 20.00 ± 3.83 | 16.86 ± 3.29 | 23.14 ± 5.49^§^ | 31.57 ± 9.00 | 25.43 ± 5.19 | 31.29 ± 6.24 |
| Mean Vel, cm/s | 36.14 ± 6.77 | 28.71 ± 4.31 | 41.57 ± 8.28^§^ | 32.57 ± 6.73 | 25.43 ± 5.59 | 40.86 ± 9.08^‡§^ | 30.00 ± 5.77 | 25.42 ± 4.86 | 36.71 ± 7.83^§^ | 39.71 ± 10.23 | 32.14 ± 6.01 | 41.43 ± 9.66 |
| **UV** |  |  |  |  |  |  |  |  |  |  |  |  |
| PI | 0.34 ± 0.04 | 0.47 ± 0.05^†^ | 0.42 ± 0.12 | 0.34 ± 0.08 | 0.41 ± 0.10 | 0.39± 0.08 | 0.30 ± 0.04 | 0.39 ± 0.05 | 0.41± 0.11 | 0.32 ± 0.03 | 0.41 ± 0.10 | 0.34± 0.03 |
| Syst Vel, cm/s | 65.25 ± 18.39 | 40.50 ± 10.66^†^ | 34.00 ± 5.71^‡^ | 56.75 ± 16.52 | 33.00 ± 9.83^†^ | 33.75 ± 9.78^‡^ | 50.25 ± 11.44 | 30.00 ± 10.23^†^ | 26.25 ± 3.30^‡^ | 70.75 ± 17.46 | 47.50 ± 11.68^†^ | 36.75 ± 4.19^‡^ |
| Diast Vel, cm/s | 45.75 ± 11.35 | 25.25 ± 7.93^†^ | 22.50 ± 5.80^‡^ | 39.50 ± 8.70 | 22.00 ± 8.21^†^ | 22.25 ± 2.25^‡^ | 37.25 ± 8.73 | 20.25 ± 7.54^†^ | 17.25 ± 1.11^‡^ | 50.75 ± 11.93 | 31.50 ± 9.95^†^ | 26.00 ± 2.83^‡^ |
| Mean Vel, cm/s | 55.50 ± 15.67 | 33.00 ± 8.91^†^ | 28.25 ± 6.08^‡^ | 48.50 ± 12.90 | 28.00 ± 9.63^†^ | 28.25 ± 8.10^‡^ | 44.00 ± 10.17 | 25.00 ± 7.53^†^ | 22.00 ± 2.71^‡^ | 61.00 ± 14.51 | 39.75 ± 11.50^†^ | 31.75 ± 2.63^‡^ |
| UA, umbilical artery; UV, umbilical vein; PI, pulsatility index; S/D, systolic/ diastolic ratio; Syst Vel, systolic velocity; Diast Vel, diastolic velocity; Mean Vel, mean velocity. Data are presented as mean ± SD. *P*-values for differences according to one-way ANOVA with a Fisher’s least significant difference (LSD) *post-hoc* test, or Kruskal-Wallis (K-W) test. †, *P* < 0.05 *vs.* distal segment (UA2/UV2 *vs.* UA1/UV1); ‡, *P* < 0.05 *vs*. distal segment (UA3/UV3 *vs.* UA1/UV1); §, *P* < 0.05 *vs.* middle segment (UA3/UV3 *vs.* UA2/UV2). | | | | | | | | | | | | |
